# Impulsivity profile analysis and it’s potential role in the differential diagnostics of adult Attention Deficit Hyperactivity Disorder and Borderline Personality Disorder

**DOI:** 10.1192/j.eurpsy.2024.497

**Published:** 2024-08-27

**Authors:** E. Kenézlői, S. Somogyi, L. Balogh, E. Lévay, B. Bajzát, Z. Halmai, Z. S. Unoka, J. Réthelyi

**Affiliations:** ^1^Psychiatry and Psychotherapy, Semmelweis University; ^2^Bhaktivedanta Collage, Budapest, Hungary

## Abstract

**Introduction:**

Impulsivity is a complex construct, having at least three factors: 1) impulsivity as a personality trait, 2) ismpulsive action – waiting and stopping impulsivity and 3) choice impulsivity. Impulsive symptoms are present in Attention Deficit Hyperactivity Disorder (ADHD) and Borderline Personality Disorder (BPD) as well, eventhough impulsvity profile significantly differs.

**Objectives:**

Our aim is to describe the impulsivity profile in adult ADHD (aADHD) and BPD in comparison with the control group, and describe a characteristic pattern, which associates with these disorders.

**Methods:**

aADHD (n=100) and BPD Patients (n=63) were included, based on DSM-5 diagnostic criteria. Healthy control subjects (n=100) were screened using the Derogatis Symptom Checklist (SCL-90). Comorbid psychiatric disorders were assessed by structured clinical interviews and those who have both aADHD and BPD were excluded from the study. Participants were further investigated with online questionnaires: e.g. Barratt Impulsiveness Scale (BIS-11) Difficulties in Emotion Regulation Scale (DERS) and neuropsychological tests, like CANTAB Rapid Visual Processing, Stop Signal Task, and the Rogers’ decision-making test.

**Results:**

Based ont the BIS-11 results, significantly higher attentional impulsivity was present in adult ADHD compared to BPD (p<.001) and healthy controls (p<.001). Emotional regulation difficulties, measured by DERS were significantly higher in BPD (p<.001) than aADHD, but the impulse control problems were more pronounced in the aADHD group, compared to BPD (p<.001). Using CANTAB neuropsychological test battery, strategy formulation difficulties (p=0.16) and stopping impulsivity (p<.001) were only present in aADHD compared to HC. BPD patients did not differ significantly from the control group in strategy formulation and in Stop Signal Reaction Time, a measure of stopping impulsivity. The significantly higher level of total false alarms, reflecting on waiting impulsivity were present both in aADHD and BPD.

**Conclusions:**

According to our results these two disorders have different impulsivity profile characteristics, which can be useful in differentiating these two disorders, and in buiding treatment plans. Stopping impulsivity, measured by SST was found in aADHD, but not in BPD. In BPD impulsive behavior is more likely attached to emotional dysregulation, a trait rooted in childhood traumatization.

This study was supported by the National Research, Development and Innovation Office grant K 129195 and K 135437.

**Disclosure of Interest:**

None Declared

